# Editorial: Modelling neurodevelopmental and neurodegenerative diseases for prognosis, diagnosis, and therapies

**DOI:** 10.3389/fcell.2024.1403645

**Published:** 2024-04-24

**Authors:** Sven Vilain

**Affiliations:** Department of Medical Biology, Health Science and Technologies Research, Institute (SABITA), Istanbul Medipol University, Istanbul, Türkiye

**Keywords:** down syndrome, fatty acid hydroxylase-associated neurodegeneration, inherited retinal disease (IRD), neural tube defects (NTD), hypoxia induced factor-1 (HIF-1), Alzheimer’ disease

The human brain is an intricate organ, central to cognition and behavior. Proper brain development and maintenance are crucial for overall wellbeing. Various factors can disrupt normal brain development, like trisomy 21 and fetal alcohol exposure, or lead to degenerative states later in life, like brain oxygen deprivation or certain genetic mutations. Specific markers are often used to help with prognosis, early diagnosis, progression and monitoring the response to treatment. Discovery of these markers require elucidating underlying pathological mechanisms and identifying associated risk factors. Pathological disease mechanisms are often examined in model organisms as they permit investigating genetic and molecular basis of fundamental biological processes. This Research Topic contains contributions describing current knowledge and novel findings in different fields of neurodevelopmental and neurodegenerative disorders. Original papers include association studies and research with *Drosophila melanogaster* and *Xenopus laevis*. In total, the Research Topic “*Modelling Neurodevelopmental and Neurodegenerative Diseases for Prognosis, Diagnosis, and Therapies*” collected three reviews and three original contributions. These contributions are divided in neurodevelopmental disorders (Down syndrome and neural tube defects), cerebral hypoxia and neurodegenerative disorders (Inherited Retinal Diseases, Alzheimer’s disease, and Fatty Acid Hydroxylase-associated Neurodegeneration), which we will describe succinctly in this editorial.

Understanding the cause and underlying mechanisms of neurodevelopment disorders is crucial to improve patient’s care and treatment. Individuals with trisomy 21 exhibit structural and functional defects in multiple systems. The extra chromosome 21 results in many congenital clinical defects such as developmental delay and neurodevelopmental disorders. The first manuscript (Hasina et al.) discusses the neuronal defects observed in human patients with Down syndrome from embryonic development to adulthood. The authors provide a comprehensive overview of the various studies on Down syndrome and describe the changes that occur at the anatomical, cellular and molecular levels. Moreover, they point out knowledge gaps and propose molecular networks to describe the mechanisms associated with Down syndrome. As such, the authors present a comprehensive summary of the changes in brain development of patients with trisomy 21.

Other neurodevelopmental disorders include neural tube defects, which are among the most common birth defects in humans, attributed to abnormal folic acid metabolism, excess retinoic acid (RA), environmental factors, and others causes. *In utero* exposure of human embryos to ethanol causes fetal alcohol spectrum disorder, which causes several developmental disorders, including neural tube defects and brain malformations. Since the mechanisms to clear alcohol and synthesize RA share overlapping enzymes, these observations suggest that a reduction in RA signaling may also cause defects in neuronal tube closure. In the next research paper (Edri et al.), the authors used *X. laevis* embryos and exposed them to compounds that inhibit RA biosynthesis, such as ethanol, or compounds that break down RA, and studied their effects on neural tube development. Using markers and morphological analysis, the authors found that reducing RA signaling shortly after the induction of neural plate precursors altered normal neural plate proliferation and differentiation. Therefore, RA signaling defects lead to increased proliferation and expansion of the neural plate.

The third article of this Research Topic (Mitroshina et al.) discusses the brain’s response to oxygen deprivation. The brain requires much oxygen as it uses a lot of energy. A lack of oxygen causes neuronal dysfunction and is often associated with neurodegenerative diseases. This manuscript discusses the hypoxia-inducible factors (HIFs) family of transcription factors and their protective role during cerebral hypoxia. The main cause of hypoxia in the brain is ischemic stroke, but it is also common in neurodegenerative disorders. Maintaining cell viability is critical in hypoxic areas of the brain and the HIF complex exerts a neuroprotective response. In this review, the authors provide an overview of HIF-1 function and the pathways activated by HIF-1 to oxygen deprivation. Furthermore, they focus on the putative role of HIF-1 in neuronal pathologies and its potential role as a novel therapeutic target. Therefore, this manuscript provides insights in the cellular responses to oxygen deprivation and neuronal loss.

Neurodegenerative diseases encompass a group of debilitating conditions characterized by progressive dysfunction and loss of neurons. Some affecting the neuronal tissue of the retina, causing Inherited retinal diseases (IRD). IRDs, caused by the degeneration of light-sensitive photoreceptors, are heterogeneous disorders that lead to progressive visual impairment and blindness. The cause of this degeneration can be direct and/or indirect via dysfunction of interacting partners in the retina. As photoreceptors interact with the retinal pigment epithelium and Müller glia, mutations in genes associated specifically with these tissues can cause IRDs as well. To better understand the defects underlying IRDs, the next manuscript (Du et al.) describes the known interactions between photoreceptors and retinal pigment epithelium/Müller glia, as well as the potential implications for disease pathogenesis when these interactions are defective. Furthermore, the authors summarize recent developments in IRD therapies targeting retinal pigment epithelium and/or Müller glia.

Association studies can unravel the genetic architecture of complex phenotypes and identify shared genetic influences that may predict disease risk. The next research manuscript examines the association between low heel bone mineral density (BMD) and Alzheimer’s disease (AD) (Gao et al.). Observational studies suggested a possible link between the two, but medical randomization studies did not appear to support this causal link. The authors considered a larger number of variables than previous studies and evaluated the causal effect of heel BMD on the risk of AD. By analyzing large-scale heel BMD and AD GWAS datasets, the authors observed a statistically significant causal effect of heel BMD on AD risk, supporting heel BMD as a risk factor for AD and suggesting a further evaluation of the heel BMD and AD association.

The final paper describes a novel fruit fly model to study Fatty Acid Hydroxylase-associated Neurodegeneration (FAHN). FAHN is a rare disease that causes neurodegeneration with accumulation of iron in the brain. The condition is caused by autosomal recessive loss-of-function mutations in fatty acid 2-hydroxylase (FA2H), a gene involved in the synthesis of many 2-hydroxylated sphingolipids. To gain new insights into the pathological mechanisms of FAHN, the last manuscript describes the generation and validation of a novel *D. melanogaster* disease model of FAHN (Mandik et al.). Flies with *dfa2h* (fruit fly homolog of *FA2H*) los-of-function mutations are viable but exhibit behavioral abnormalities and a shortened lifespan. At the cellular level, these flies exhibit altered mitochondrial dynamics and defects in autophagy. These defects are possibly evolutionary conserved as patient-derived fibroblasts exhibit similar defects. Therefore, the authors established and validated a *Drosophila* disease model, allowing further investigation into the cellular mechanism of FAHN.

It is anticipated that the articles in this Research Topic will significantly advance our comprehension of neuronal disorders and pave the way for enhanced treatment methodologies.

